# Anti-cancer effect of LINC00478 in bladder cancer correlates with KDM1A-dependent MMP9 demethylation

**DOI:** 10.1038/s41420-022-00956-z

**Published:** 2022-05-03

**Authors:** Han-Jie Yang, Tian Liu, Yang Xiong

**Affiliations:** Department of Urology, Pingxiang Affiliated Hospital, Southern Medical University, 337000 Pingxiang, P. R. China

**Keywords:** Cancer, Cell biology

## Abstract

Accumulating evidence has highlighted the important roles of long intergenic non-coding RNAs (lincRNAs) during cancer progression. However, the involvement of LINC00478 in bladder cancer remains largely unclear. Accordingly, the current study sought to investigate the function of LINC00478 on malignant phenotypes of bladder cancer cells as well as the underlying mechanism. By integrating data from in silico analysis, we uncovered that LINC00478 was differentially expressed in bladder cancer. We further analyzed the expression of LINC00478 and matrix metalloprotein 9 (MMP9) in bladder cancer tissues and cell lines and observed a significant decline in LINC00478 expression and an elevation in MMP9 expression. In addition, chromatin immunoprecipitation, RNA-binding protein immunoprecipitation, and RNA pull-down assays predicted and validated that LINC00478 targeted lysine-specific demethylase-1 (KDM1A) and down-regulated the expression of MMP9 by decreasing the monomethylation on lysine 4 of histone H3 (H3K4me1) of MMP9 promoter. Treatment with KDM1A inhibitor tranylcypromine (TCP) also led to an increase in the enrichment of H3K4me1 in the MMP9 promoter region. Through gain- and loss-of-function approaches, we found that LINC00478 up-regulation diminished the malignant phenotype of bladder cancer cells in vitro, and further inhibited xenograft tumor growth and metastasis in vivo by repressing MMP9. Collectively, our findings unraveled a LINC00478-mediated inhibitory mechanism in bladder cancer *via* the recruitment of histone demethylation transferase KDM1A to the MMP9 promoter region, which can provide potential implications for novel therapeutic targets against bladder cancer.

## Introduction

Bladder cancer is a highly complex disease leading to alarming morbidity and death rates, accounting for approximately 170,000 deaths across the globe on an annual basis [1, 2]. Further adding to the plight, metastatic bladder cancer is associated with dismal prognoses, with a 5-year survival rate of less than 10% [3]. Unfortunately, bladder cancer has a high tendency to metastasize to numerous sites, such as the brain, lung, as well as bone, while the underlying mechanisms of its development and metastasis remain poorly understood [4].

A subset of noncoding transcripts termed as long noncoding RNAs (lncRNAs), which contain at least 200 nucleotides [5], are well-established to exhibit indispensable roles in the modulation of cellular growth and differentiation. Moreover, the manipulation of lncRNA expression is known to influence the progression of various cancers [6]. What is noteworthy, prior evidence has documented that the tissue- and cancer-specific expression of lncRNAs can function as potential markers in urologic malignancies, including bladder cancer [7]. Interestingly, the expression profiles in The Cancer Genome Atlas (TCGA) retrieved by Zhang et al. [8] previously indicated that LINC00478 is differentially expressed in breast cancer, and further highlighted the diagnostic value of LINC00478 in regard to breast cancer. Bioinformatics analysis of the Gene Expression Omnibus (GEO) database further indicates that LINC00478 was poorly expressed in bladder cancer. On another note, matrix metalloproteinases (MMPs) have been identified as a key player in tumor invasion and metastasis [9]. Formerly described transcriptional controlling of MMP9 is comprised of epigenetic mechanisms, including histone modifications and microRNAs (miRNAs) [10]. In addition, recent investigations have highlighted the inhibitory effect of miR-300 exerted on bladder cancer cell metastasis by virtue of down-regulating MMP9 [11]. Furthermore, a prior study highlighted that lysine-specific demethylase-1 (KDM1A) inhibition could contribute to the elevation of monomethylation on lysine 4 of histone H3 (H3K4me1) [12].

In lieu of the preceding evidence, we speculated the involvement of LINC00478 in bladder cancer progression via the mediation of KDM1A-dependent MMP9 demethylation. Accordingly, the current study set out to explore the regulatory mechanism underlying LINC00478 functioning in the modulation of malignancy and metastasis in bladder cancer cells.

## Results

### LINC00478 is poorly expressed in bladder cancer tissues and cells

**Fig. 1:**
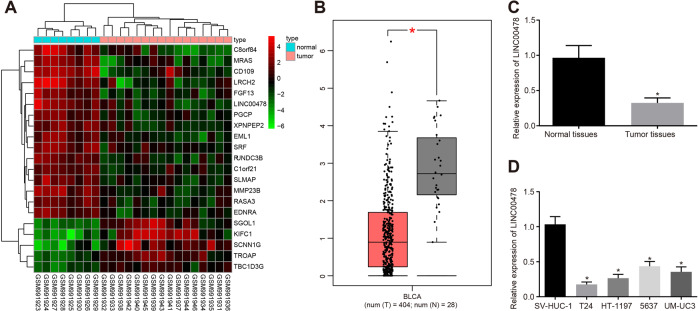
LINC00478 is poorly expressed in bladder cancer. **A** The heatmap showing differentially expressed lncRNAs in bladder cancer expression profile GSE40355. The horizontal coordinates indicate sample numbers, the vertical coordinate indicates differentially expressed genes, and the upper right histogram indicates color scale in which each rectangle corresponds to the expression of a sample. **B** The prediction of the LINC00478 expression in normal tissues and bladder cancer tissues according to GEPIA website. Red indicates the tumor tissue, and gray indicates the normal tissue. **C** LINC00478 expression in normal bladder epithelial tissues and bladder cancer tissues measured by RT-qPCR (*n* = 72). **D** The expression of LINC00478 in the normal bladder epithelial cell line (SV-HUC-1) and bladder cancer cell lines (T24, UM-UC3, HT-1197, and 5637) determined by RT-qPCR. *p < 0.05 vs. normal bladder epithelial tissues or SV-HUC-1 cells. Measurement data were described as mean ± standard deviation. The paired *t* test was used for comparison between two groups, and one-way ANOVA was used for comparison among multiple groups, followed by Tukey’s post hoc test. The cell experiment was repeated three times independently.

Differential analysis of the microarray dataset GSE40355 (Fig. 1A) and GEPIA website (http://gepia.cancer-pku.cn/index.html) (Fig. 1B) suggested that LINC00478 expression was diminished in bladder cancer. Subsequently, LINC00478 expression patterns were evaluated in clinically-collected bladder cancer tissues and commercially-purchased cells. The results of RT-qPCR demonstrated that LINC00478 expression was significantly reduced in bladder cancer tissues versus that in normal bladder epithelial tissues (Fig. 1C). In addition, LINC00478 expression was profoundly diminished in all tested human bladder cancer cell lines (T24, UM-UC3, HT-1197, and 5637) relative to the normal human bladder epithelial cell line SV-HUC-1 (Fig. 1D). Among all the bladder cancer cell lines included, LINC00478 expression was the lowest in the T24 cells and the highest in 5637 cells, and consequently these two cell lines were selected for further experimentation.

We subsequently analyzed the correlation between LINC00478 expression and clinic-pathological factors in 72 patients with bladder cancer. As shown in Supplementary Table 1, LINC00478 expression was negatively correlated with histological grade and Tumor, Node, Metastases (TNM) stage. Meanwhile, clinic factors such as patients’ age, gender, and lymph node metastasis (LNM) were found not to be associated with LINC00478 expression.

Taken together, these findings validated that the expression of LINC00478 was frequently decreased in bladder cancer.

### LINC00478 inhibits malignant phenotypes of bladder cancer cells

**Fig. 2:**
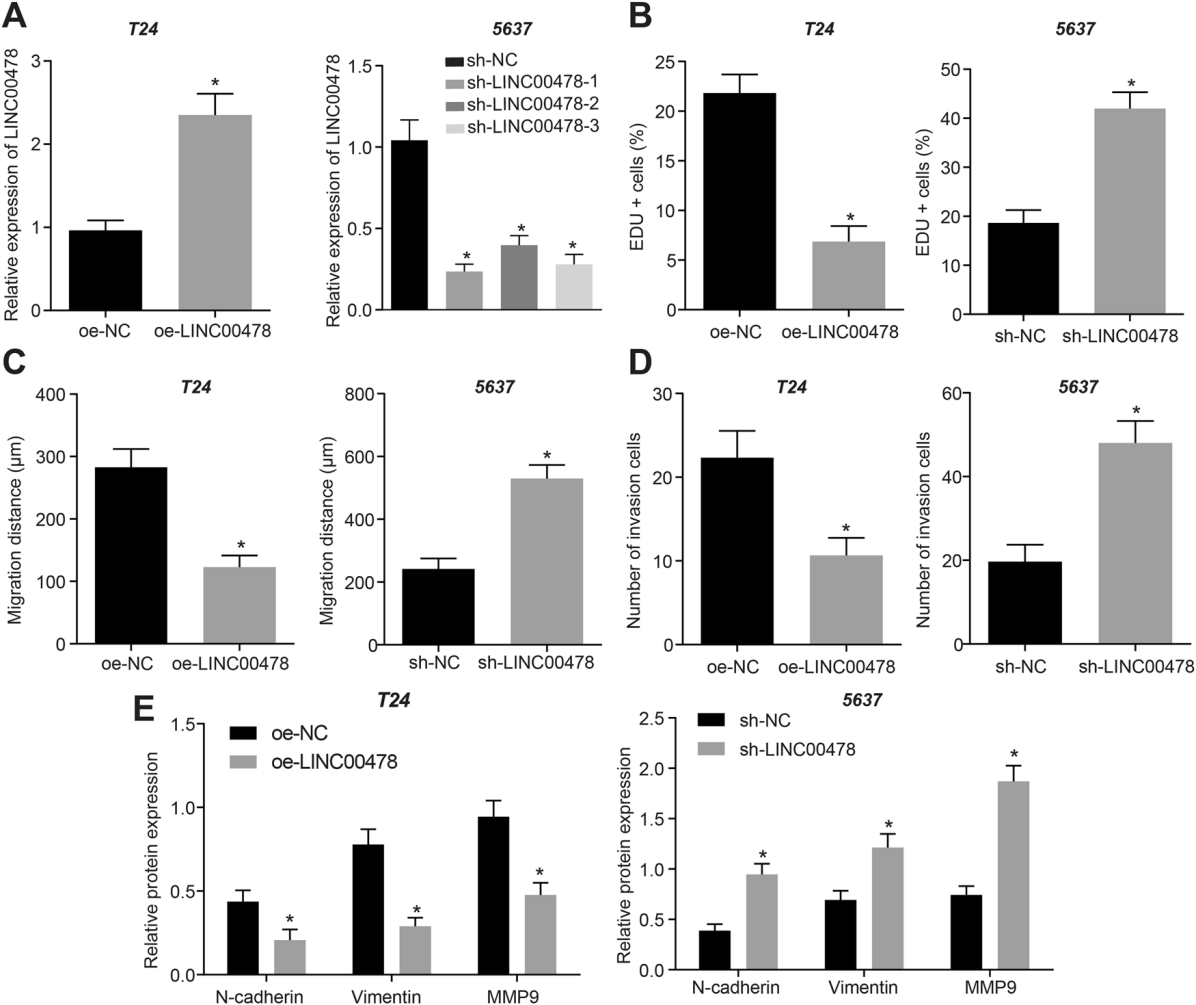
Over-expression of LINC00478 inhibits proliferation, migration, and invasion of bladder cancer cells. T24 cells were transfected with oe-NC or oe-LINC00478, while 5637 cells are treated with sh-NC or sh-LINC00478. **A** The expression of LINC00478 in T24 and 5637 cells evaluated with RT-qPCR. **B** The cell proliferation tested by EdU assay. **C** The cell migration assessed by wound healing assay. **D** The cell invasion determined by Transwell assay. **E** The expression of N-cadherin, Vimentin and MMP9 measured by Western blot. *p < 0.05 vs. T24 cells treated with oe-NC or 5637 cells treated with sh-NC. Measurement data were described as mean ± standard deviation. The independence sample *t* test was used for comparison between two groups, and one-way ANOVA was used for comparison among multiple groups, followed by Tukey’s post hoc test. Cell experiment was repeated 3 times independently.

To further investigate the effects of LINC00478 on bladder cancer development, we over-expressed LINC00478 in the T24 cell line, a bladder cancer cell line with the lowest LINC00478 expression, whereas the bladder cancer cell line 5637 with the highest LINC00478 expression was treated with shRNA-1 against LINC00478 (sh-LINC00478-1), sh-LINC00478-2 or sh-LINC00478-3. Over-expression and silencing efficiency of LINC00478 was validated by RT-qPCR. The expression of LINC00478 in T24 cells treated with over-expressed LINC00478 (oe-LINC00478) was elevated relative to those with negative control for gene over-expression (oe-NC), whereas LINC00478 expression following sh-LINC00478-1, sh-LINC00478-2, sh-LINC00478-3 treatment was significantly reduced versus NC for shRNA (sh-NC) treatment; wherein sh-LINC00478-1 exhibited the most profound silencing efficiency. Therefore, sh-LINC00478-1 was selected for the subsequent experimentation (Fig. 2A). Thereafter, the effects of LINC00478 on cell proliferation were detected with EdU assay, and the results illustrated that LINC00478 up-regulation repressed the proliferation of bladder cancer cell line T24, while silencing LINC00478 promoted the proliferation of bladder cancer cell line 5637 (Fig. 2B and Supplementary Fig. 1A). Meanwhile, wound healing and Transwell assay results displayed that LINC00478 up-regulation suppressed the migration and invasion of T24 cells, whereas silencing LINC00478 boosted those in the bladder cancer cell line 5637 (Fig. 2C, D and Supplementary Fig. 1B, C).

Furthermore, the results of Western blot illustrated that the expression of N-cadherin, Vimentin, and MMP9 was decreased in bladder cancer cells after over-expression of LINC00478 in T24 cells while being elevated following LINC00478 silencing in 5637 cells (Fig. 2E and Supplementary Fig. 1D).

Altogether, these findings revealed that LINC00478 up-regulation inhibited the bladder cancer cell malignant phenotype while silencing LINC00478 led to the opposite trends.

### MMP9 expresses at high levels in bladder cancer cells

**Fig. 3:**
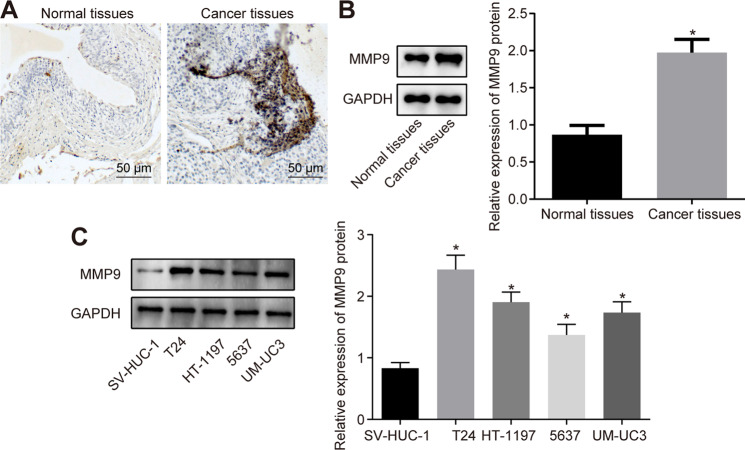
High expression of MMP9 in bladder cancer tissues and cells is identified. **A** The expression of MMP9 in bladder cancer and normal bladder epithelial tissues detected by immunohistochemistry. **B** MMP9 expression in bladder cancer and normal bladder epithelial tissues determined by Western blot (*n* = 72). **C** MMP9 expression in bladder cancer cells and normal bladder epithelial cells measured by Western blot. *p < 0.05 vs. normal bladder epithelial tissues or SV-HUC-1 cells. Measurement data were described as mean ± standard deviation. The paired *t* test was used for comparison between two groups, and one-way ANOVA was used for comparison among multiple groups, followed by Tukey’s post hoc test. The cell experiment was repeated three times independently.

After uncovering that over-expression of LINC00478 impaired the MMP9 expression in bladder cancer cells, we focused our efforts on investigating whether LINC00478 affects the bladder cancer cell malignant phenotype by modulating the expression of MMP9. The results of Immunohistochemical staining illustrated higher expression of MMP9 in bladder cancer tissues compared to that in normal bladder epithelial tissues (Fig. 3A), and the same was validated by Western blot (Fig. 3B). In addition, MMP9 expression was enhanced in bladder cancer cells (T24, UM-UC3, HT-1197, and 5637) relative to that in normal bladder epithelial cells (SV-HUC-1), with T24 cells exhibiting the highest expression (Fig. 3C).

These findings demonstrated that MMP9 expression was increased in bladder cancer tissues and cells.

### LINC00478 promotes histone demethylation of MMP9 by recruiting KDM1A

**Fig. 4:**
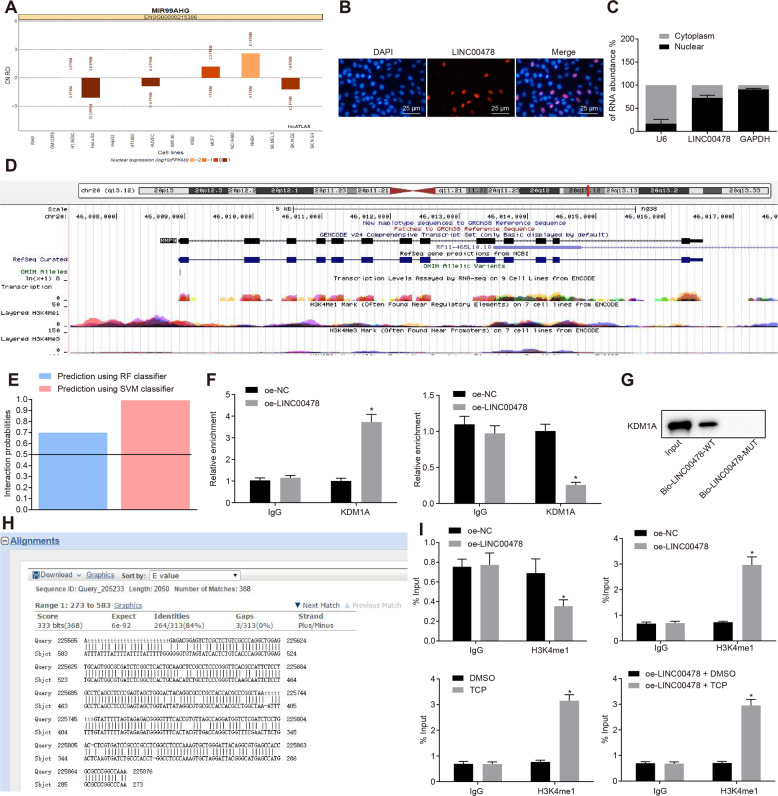
LINC00478 recruits KDM1A to increase MMP9 histone demethylation. **A** The prediction of LINC00478 localization by lncATLAS website. **B** LINC00478 subcellular localization detected by FISH assay. **C** LINC00478 subcellular localization detected by nuclear/cytoplasmic fractionation assay. **D** The enrichment of high-level H3K4me1 and low-level H3K4me3 in the MMP9 promoter region revealed by the UCSC website. The peak indicates the expression level. **E** The analysis of binding relationship between LINC00478 and histone H3K4 demethylase KDM1A by RPISeq website in which Random Forest (RF) and Support Vector Machine (SVM) > 0.5 indicates there is a binding relationship. Prediction using RF classifier = 0.85. Prediction using SVM classifier = 0.99. **F** The binding between LINC00478 and KDM1A detected by RIP. **G** KDM1A pulled-down by LINC00478 detected by RNA pull-down assay. **H** Blast comparison of LINC00478 and MMP9 promoter sequences. **I** The analysis of H3K4me1 enrichment in the promoter region of MMP9 by ChIP assay. *p < 0.05 *vs*. bladder cancer cells treated with oe-NC, sh-NC, DMSO or oe-LINC00478 + DMSO. Measurement data were described as mean ± standard deviation. The independence sample *t* test was used for comparison between the two groups. The cell experiment was repeated three times independently.

Previously published literature suggests that MMP9 can be epigenetically regulated, including histone modification and miRNA regulation [10], and therefore we decided to explore whether LINC00478 down-regulated MMP9 through epigenetic regulation. Analyses from the LncATLAS website suggested that LINC00478 was primarily localized in the nucleus (Fig. 4A), and the same was confirmed by fluorescent in situ hybridization (FISH) and Nuclear/Cytoplasmic fractionation assays (Fig. 4B, C). Meanwhile, the UCSC website (http://genome.ucsc.edu/) indicated that H3K4me1 was highly enriched in the MMP9 promoter region (Fig. 4D), suggesting that histone H3K4me1 modification of MMP9 promoter may be an important reason for regulating the expression of MMP9.

In order to further investigate how LINC00478 regulated MMP9 expression, firstly, the RPISeq website (http://pridb.gdcb.iastate.edu/RPISeq/) was adopted to predict potential binding between LINC00478 and histone H3K4 demethylase KDM1A (Fig. 4E). Moreover, RIP data illustrated that depletion of LINC00478 decreased the binding of KDM1A, whereas restoration of LINC00478 brought about the opposite trends in KDM1A binding (Fig. 4F). Additionally, the results of RNA pull-down assay demonstrated that KDM1A protein was pulled down by Bio-LINC00478-wild type (wt), but not by Bio-LINC00478-mutant (mut), highlighting that LINC00478 recruited histone H3K4 demethylase KDM1A (Fig. 4G).

Furthermore, the Basic Local Alignment Search Tool (Blast) revealed the presence of complementary base pairing between LINC00478 and MMP9 promoter in the form of RNA-DNA (Fig. 4H). In addition, the results of ChIP assay for enrichment of H3K4me1 in the MMP9 promoter region demonstrated that knockdown of LINC00478 increased the enrichment of H3K4me1 in the MMP9 promoter region, while over-expression of LINC00478 led to the opposing trends in H3K4me1 enrichment at the MMP9 promoter region. Moreover, treatment with KDM1A-specific inhibitor tranylcypromine (TCP) resulted in the profound promotion of H3K4me1 enrichment at the MMP9 promoter and was further elevated by over-expression of LINC00478 (Fig. 4I).

Altogether, these findings indicated that LINC00478 reduced histone methylation of MMP9 promoter by recruiting KDM1A, thus diminishing the expression of MMP9.

### LINC00478 inhibits malignant phenotypes of bladder cancer via the suppression of MMP9 expression

**Fig. 5:**
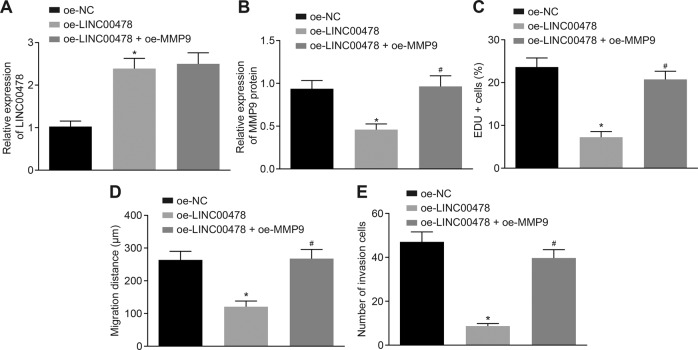
Bladder cancer cell proliferation, invasion, and migration are repressed by LINC00478 via inhibition of MMP9. T24 cells are transfected with oe-LINC00478 alone or with oe-MMP9. **A** LINC00478 expression in T24 cells determined by RT-qPCR. **B** MMP9 expression measured by Western blot. **C** The cell proliferation tested by EdU assay. **D** The cell migration assessed by wound healing assay. **E** The cell invasion determined by transwell assay. *p < 0.05 vs. T24 cells treated with oe-NC. ^#^*p* < 0.05 vs. T24 cells treated with oe-LINC00478. Measurement data were described as mean ± standard deviation. The one-way ANOVA was used for comparison among multiple groups, followed by Tukey’s post hoc test. The cell experiment was repeated three times independently.

In an effort to explore the effect of LINC00478 on the MMP9 expression in bladder cancer, we over-expressed LINC00478 alone or with MMP9 in T24 cells. Subsequently, the results of RT-qPCR illustrated that LINC00478 expression was significantly increased in response to oe-LINC00478 in T24 cells, yet further treatment of oe-MMP9 did not markedly alter the LINC00478 expression (Fig. 5A). Meanwhile, Western blot indicated that MMP9 expression exhibited a significant decline after LINC00478 was over-expressed in T24 cells, which was rescued by co-expressing MMP9 (Fig. 5B and Supplementary Fig. 2A).

Moreover, over-expression of LINC00478 weakened the proliferation, migration, and invasion abilities of T24 cells, and these inhibitory effects were reversed by restoration of MMP9 (Fig. 5C–E and Supplementary Fig. 2B–D).

Overall, these findings indicated that LINC00478 up-regulation represses the bladder cancer cell malignant phenotype by inhibiting MMP9.

### LINC00478 inhibits bladder cancer development and metastasis in vivo by decreasing MMP9 expression

**Fig. 6:**
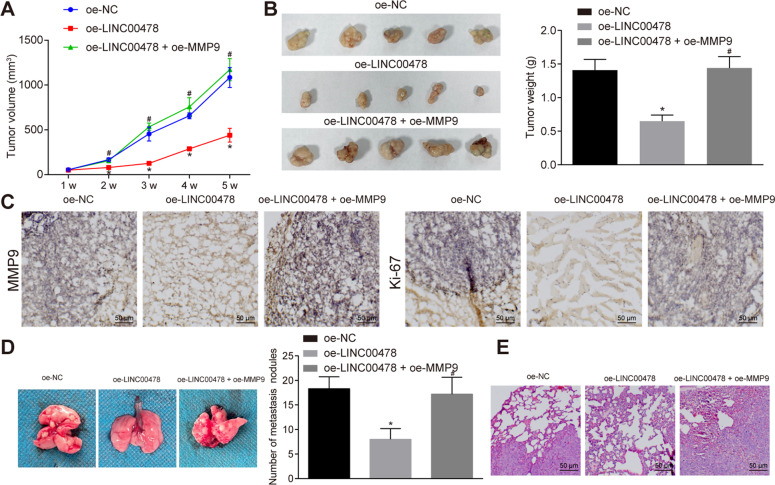
Over-expression of LINC00478 inhibits growth and metastasis of bladder cancer cells in vivo by inhibiting MMP9. **A** The growth curve of transplanted tumor in mice of each group (*n* = 15). **B** Representative images of tumors and tumor weight in nude mice (*n* = 15). **C** The expression of MMP9 and Ki67 in transplanted tumors detected by immunohistochemistry. **D** The number of lung nodules in metastatic animal models (*n* = 5). **E** The morphological changes of lung tissues observed by H&E staining. *p < 0.05 vs. nude mice harboring T24 cells treated with oe-NC. ^#^*p* < 0.05 vs. nude mice harboring T24 cells treated with oe-LINC00478. Measurement data were described as mean ± standard deviation. The one-way ANOVA was used for comparison among multiple groups, followed by Tukey’s post hoc test, and the repeated measures ANOVA was applied for the comparison of data at different time points with Bonferroni’s post hoc test.

Furthermore, we investigated the effects of LINC00478 and MMP9 on the growth of bladder cancer in vivo. Following the injection of stably-transfected T24 cells into nude mice, tumor volume and weight of nude mice were analyzed and found to be diminished in the oe-LINC00478-treated mice versus the oe-NC-treated mice (Fig. 6A, B). The aforementioned effects were partly reversed by treatment with oe-LINC00478 + oe-MMP9. The expression of MMP9 (localized in the extracellular matrix) and Ki67 (localized in the nucleus) was evaluated using immunohistochemistry, the results of which exhibited profound declines in MMP9 and Ki67 expression in oe-LINC00478-treated mice compared to oe-NC-treated mice (Fig. 6C).

Lastly, an in vivo tumor metastasis test was carried out to study the effect of LINC00478 and MMP9 on the metastatic capacity of bladder cancer cells. Subsequent findings illustrated that the number of lung nodules in the oe-LINC00478-treated mice was reduced relative to that in the oe-NC-treated mice while being increased in the mice treated with oe-LINC00478 + oe-MMP9 relative to mice treated with oe-LINC00478 alone (Fig. 6D). Moreover, H&E staining demonstrated an alleviated degree of pathological changes in oe-LINC00478-treated mice, which was aggravated in mice treated with oe-LINC00478 + oe-MMP9 (Fig. 6E).

Collectively, the above-mentioned findings revealed that over-expression of LINC00478 hindered bladder cancer growth and metastasis in vivo through inhibition of MMP9.

## Discussion

The hard-done work of our peers has shed a light on the importance of lncRNAs in tumorigenesis of bladder cancer [13], and further paved the way for studies like ours to uncover the underlying mechanisms of lncRNAs in the same. Similarly, lncRNAs are known to exhibit distinct expression patterns in primary tumor progression and metastases, and also possess the ability to bind to chromatin modification complexes or proteins to modulate gene expression [14]. Herein, we uncovered the expression of LINC00478 was significantly reduced in bladder cancer tissues relative to that in adjacent normal bladder epithelial tissues. Subsequently, we found that LINC00478 diminished the MMP9 expression *via* KDM1A recruitment in bladder cancer. Furthermore, enforced expression of LINC00478 in bladder cancer tissues was implicated to inhibit bladder cancer cell malignant phenotype in vitro and suppress tumorigenicity in vivo.

Initially, our findings evidenced that LINC00478 was poorly expressed, while MMP9 was highly expressed in bladder cancer. Microarray analyses of lncRNAs expression profiles have previously offered valuable insight into the illumination of the roles of lncRNAs in human cancers [15]. Likewise, we investigated the aberrant lncRNA expression patterns in bladder cancer by microarray analysis and recognized a novel lncRNA LINC00478. Subsequent experiments validated that LINC00478 was down-regulated in bladder cancer tissues compared to adjacent normal bladder epithelial tissues. Meanwhile, LINC00478 was previously documented to be abnormally expressed in breast cancer, and exhibit potential implications in modulating the progression of breast cancer [16]. Also notably, a prior study highlighted the association between LINC00478 and tumor differentiation and lymphatic metastasis in patients with vulvar squamous cell carcinoma [17].

It is also interesting that a prior bioinformatics investigation indicated the involvement of LINC00478 in breast cancer [8], but failed to illuminate the specific mechanistic actions and downstream factors. Herein, we uncovered that ectopic expression of LINC00478 suppressed the malignant phenotypes of bladder cancer cells. More importantly, our findings identified that LINC00478 inhibited the expression of MMP9 by recruiting the histone demethylase KDM1A to the MMP9 promoter region. KDM1A has also been reported to suppress or induce the transcription by controlling histone H3K4me1/2 or H3K9me1/2 demethylation, respectively [18]. Additionally, mechanistic investigations carried out by Zang et al. highlighted a similar mechanism wherein LINC01133 interacted with KDM1A and recruited KDM1A to the promoter regions of Krüppel like factor 2, P21, or E-cadherin to inhibit their transcription, which is much in accordance with our data [19]. Further in line with our findings, another prior study revealed that KDM1A was recruited by homeobox A11 antisense, a lncRNA, which performed as a scaffold [20]. Moreover, KDM1A is also capable of sustaining oncogenic gene expression and impairing differentiation in a number of acute myeloid leukemia subtypes, underscoring its potential therapeutic implications in leukemia [21].

Additionally, our data unveiled that silencing of MMP9 exerted a repressive effect on bladder cancer cell malignant phenotype in vitro, and also diminished tumor growth and lung metastasis in vivo. The aforementioned MMPs, particularly MMP9, are widely-used to monitor to target tumor metastasis *via* several signaling pathways [22]. Moreover, silencing MMP9 leads to the suppression of migration in bladder cancer cells, indicating that interruption of the basement membrane by MMP9 plays an indispensable role in promoting bladder cancer cell motility and migration [23]. Meanwhile, metastasis is well-established as the primary cause of cancer-related deaths in bladder cancer, and EMT, characterized by impairment of epithelial features and restoration of mesenchymal characteristics, is known to contribute to metastasis and invasion of bladder cancer [24]. In addition, activation of EMT-related genes such as Vimentin is indicative of normal responses of basal muscle-invasive bladder cancer to interventions [25]. More importantly, data provided by Ling et al. suggested that demethylation of MMP9 promoter could play a vital role in podocyte EMT in diabetic nephropathy [26]. More so, demethylation of MMP9 was previously implicated in trophoblast cell migration, which is in line with our findings [27].

**Fig. 7:**
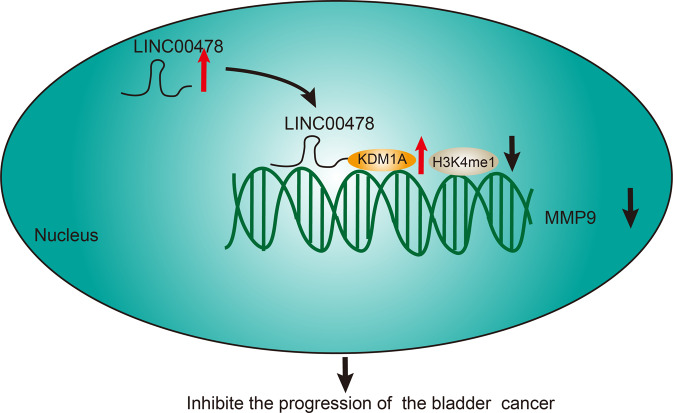
The schematic graph showing the regulatory mechanism of LINC00478 in bladder cancer. LINC00478 recruits histone demethylation transferase KDM1A to the MMP9 promoter region and diminishes the level of H3K4me1, thereby reducing the expression of MMP9 and inhibiting the progression of bladder cancer.

In conclusion, our findings established that LINC00478 was diminished in bladder cancer. On the other hand, restoration of LINC00478 exerted tumor-suppressive functions on bladder cancer by reducing cell malignant phenotype, as well as diminishing tumor growth and lung metastasis in vivo. Furthermore, LINC00478 is capable of suppressing the progression of bladder cancer through down-regulation of MMP9 by interacting with KDM1A (Fig. 7). We hope our data shed light on the potential mechanisms related to lncRNA in the regulation of bladder cancer development, and further highlights LINC00478 as a promising therapeutic target for bladder cancer treatment.

## Material and methods

### Bioinformatics analysis

The GSE40355 expression profile and its annotation probe file were retrieved from the GEO database. Differential analysis of transcriptome profiling data was subsequently carried out with the empirical Bayesian method using Bioconductor-based “limma” package from the R software. Probes were regarded as differentially expressed genes with |log fold change| > 2 and *p* values < 0.05, and the R language “pheatmap” package was adopted to plot the heatmap for differentially expressed LINC. The binding between lncRNA and protein was predicted using the RPISeq website, and the possibility of RPISeq-based interaction ranged between 0 and 1. In the performance evaluation assay, prediction with probability >0.5 was regarded as positive, indicative of a potential interaction between corresponding RNA and protein. In the cross-validation assay on the benchmark dataset, the accuracy rate of the classifier was 87–90% in accordance with the threshold value. When the classifier was tested for independent (blind) dataset, the accuracy rate of the classifier was 57–99%.

### Clinical sample collection

Bladder cancer tissues and matched adjacent normal bladder epithelial tissues were collected from a total of 72 patients with bladder cancer (including 51 males and 21 females, aged: 47–68 years, with a mean calculated age of 60.21 ± 5.37 years) hospitalized at the Pingxiang Affiliated Hospital, Southern Medical University between April 2017 and February 2019. All included cases were pathologically confirmed as bladder cancer [28]. Patients presenting with severe malnutrition, other tumors, cardiac and pulmonary dysfunction, or taking immunosuppressants, hormones, or blood products recently, or receiving radiotherapy, chemotherapy, immunotherapy, or other related treatment were excluded.

### Immunohistochemical staining

The obtained tissue sections were dewaxed, hydrated, and immersed in a water bath with a citrate retrieval solution. Tissue sections were subsequently blocked with normal goat serum blocking solution. Afterward, the sections were probed at 4 °C overnight with primary antibody, rabbit anti-MMP9 (ab194316, dilution ratio of 1:500, Abcam), and Ki67 (ab197234, dilution ratio of 1:100, Abcam), and then probed with the secondary antibody, goat anti-rabbit immunoglobulin G (IgG) (ab6785, dilution ratio of 1:1000, Abcam) at 37 °C for 20 min. Next, the sections were treated with HRP-labeled streptomyces ovalbumin working solution and developed with DAB. Following washing, the sections were counterstained with hematoxylin, blued, dehydrated, permeabilized, mounted, and observed under a microscope.

### Cell culture

Bladder cancer cell lines (namely, T24, UM-UC3, HT-1197, and 5637) and normal bladder epithelial cells (SV-HUC-1) (all procured from ATCC; Manassas, VA) were exposed to Dulbecco’s Modified Eagle Medium (DMEM; Invitrogen Inc., Carlsbad, CA, USA) containing 7% fetal bovine serum (FBS; Gibco, Carlsbad, CA, USA) and 10 µg/mL streptomycin in a 5% CO_2_ incubator (Invitrogen) at 37 °C. Upon achieving 75% confluence, the aforementioned cells were treated with different plasmids using the Lipofectamine 2000 reagent (Invitrogen). sh-LINC00478 was designed according to the Genetic Perturbation Platform (http://www.broadinstitute.org/rnai/public/) and synthesized by Shanghai Sangon. The plasmid used for loss-of-function, namely pGPU6/Neo (C02003), was purchased from Shanghai GenePharma, while that for gain-of-function, namely pCND3.1-FLAG-LPA2 (P1224) was commercially obtained from Miaolingbio Technology (Wuhan, China). After 48 h, the cells were collected for follow-up analysis. TCP (Sigma-Aldrich), a KDM1A specific inhibitor, was adopted to block the KDM1A expression in cells, with dimethylsulfoxide (DMSO) serving as the control.

### RNA isolation and quantification

Total RNA was isolated from the above-mentioned cells using the TRIzol reagent (15596026, Invitrogen) and reverse-transcribed into cDNA using the PrimeScript RT reagent kit (RR047A, Takara Biotechnology). RT-qPCR was carried out using a Fast SYBR Green PCR kit (Applied Biosystems, Inc., Foster City, CA, USA) with the ABI7500 qPCR instrument (Applied Biosystems). GAPDH was adopted as an internal control. The primers used are listed in Supplementary Table 2. Fold changes were calculated using the 2^−ΔΔCt^ method.

### Western blot

Cells in each group were collected and lysed with an enhanced RIPA lysis buffer containing protease inhibitors. The protein concentration was determined using the BCA kit. Subsequently, the protein was electrophoresed and transferred onto PVDF membranes. Following blocking with 5% silk milk for 1 h, the membrane was incubated with the following primary rabbit antibody: MMP9 (ab194316, dilution ratio of 1:1000, Abcam) or GAPDH (ab181602, dilution ratio of 1:5000, Abcam) at 4 °C overnight, and then with the secondary antibody, goat anti-rabbit IgG (ab205718, dilution ratio of 1:10,000, Abcam) at 37 °C for 1 h. The protein expression was measured with enhanced chemiluminescence and quantified using the Quantity One software.

### EdU assay

Cells were inoculated into 24-well plates and added with 10 µmol/L EdU (C10341-1, Guangzhou RiboBio). Following 2-h culture, the cells were fixed and then treated with PBS containing 0.5% Triton-100 for 20 min. Subsequently, the cells were incubated at room temperature with Apollo^®^ 567 (100 µL/well) for 30 min in conditions void of light, stained with 1× Hoechst33342 staining solution for 30 min, and mounted. The number of positive cells (stained in red) was observed and recorded using a FM-600 fluorescence microscope [29].

### Wound healing assay

Straight lines were created on the bottom of a 6-well plate using a ruler and a marker, with at least five lines in each well at an interval of 0.5–1 cm. Approximately 5 × 10^4^ cells were plated in each well and cultured overnight in DMEM containing 10% FBS. The following day, an aseptic 10 µL micropipette tip was used to create cross wounds. Wound healing was observed after a 48-h culture period. The migration of cells in each group was observed under an inverted microscope [9].

### Transwell assay

Matrigel was coated on the surface layer of the Transwell chambers at 37 °C for 30-min solidification, and the base membrane was hydrated before use. Cells in each group were resuspended in a serum-free medium at a concentration of 1 × 10^5^ cells/mL. Subsequently, 100 µL cell suspension was inoculated onto the upper surface of the membrane. Next, 10% FBS-containing culture medium was added to the basolateral chamber for 24-h incubation at 37 °C. Cells failing to invade were carefully removed, fixed, and stained. Photographs of stained invasive cells were obtained under an inverted optical microscope, and 5 fields of view were randomly chosen for cell counting.

### Fluorescent in situ hybridization

Subcellular localization of LINC00478 was detected using the Ribo^TM^ lncRNA FISH Probe Mix (Red) (RiboBio) in accordance with the manufacturer’s instructions. The LINC00478 probe was synthesized based on the LINC00478 sequence. Cells were seeded into slides in a 6-well plate and cultured for 1 day. Upon achieving 80% confluence, the cells were fixed, treated with 2 μg/mL proteinase K, glycine, and phthalation reagent, and then subjected to 1-h prehybridization with 250 μL prehybridization solution at 42 °C. Subsequently, the cells were hybridized overnight at 42 °C with hybridization solution (250 μL) containing 300 ng/mL LINC00478 probe. Five different fields were selected, viewed, and photographed using a fluorescence microscope [30].

### Nuclear/cytoplasmic fractionation

This experiment was conducted using PARIS kit (Life Technologies, Carlsbad, CA, USA) in accordance with the manufacturer’s instructions [31].

### RIP assay

Cells were initially rinsed with prechilled PBS and lysed in RIPA buffer. The supernatant was harvested, a portion of which was used as the Input, and the other was incubated with antibody for co-precipitation. Afterward, immunoprecipitated RNA were isolated and analyzed by qPCR detection. The antibody used in RIP was rabbit anti KDM1A (dilution ratio of 1:100, ab17721, Abcam) and IgG (dilution ratio of 1:100, ab109489, Abcam) as NC [32].

### RNA pull-down assay

Bladder cancer cells were transfected with biotinylated LINC00478 wt and mut (50 nM for each). The cell lysate was incubated with M-280 streptavidin magnetic beads. The expression pattern of KDM1A was examined by Western blot.

### ChIP

Cells were treated with 1% formaldehyde and incubated for 10 min to generate cross-linking. The cell lysates were subsequently sonicated to generate chromatin fragments, and immunoprecipitated overnight 4 °C with rabbit anti-H3K4me1 (dilution ratio of 1:100, ab8895, Abcam). The precipitated chromatin DNA was analyzed by qPCR.

### Tumor formation and metastasis in nude mice

A total of 45 female nude mice of SPF grade (aged 5 weeks, weighing 18–20 g) were procured for animal experimentation. Next, 6 × 10^6^ T24 cells transfected with oe-NC, oe-LINC00478 or oe-LINC00478 + oe-MMP9 were mixed with Matrigel at the ratio of 1:1, and then implanted subcutaneously in the right axillary of nude mice (0.2 mL suspension). The volume of the tumor was observed and calculated with the following formula: volume (mm^3^) = (tumor width^2^ × tumor length)/2. After 5 weeks of inoculation, all mice were euthanized with CO_2_ inhalation, and the tumor was excised and weighed. The expression of MMP9 and Ki67 was detected by immunohistochemistry.

A total of 2 × 10^6^ T24 cells stably transfected with oe-NC, oe-LINC00478, or oe-LINC00478 + oe-MMP9 were injected into the caudal vein of female thymus-free BALB/c nude mice, with 5 mice in each group. Eight weeks after injection, the nude mice were euthanized as above-mentioned, and all lungs were collected.

### H&E staining

Lung tissues were sectioned, dewaxed, and stained for 10 min with hematoxylin. Next, the samples were hydrolyzed with 1% hydrochloric acid alcohol for 30 s, and stained for 1 min with eosin. Following dehydration with gradient alcohol, the samples were permeabilized and sealed prior to observation under a Zeiss fluorescence microscope.

### Statistical analysis

Statistical analysis was carried out using the SPSS 21.0 software, with the results presented as mean ± standard deviation. Statistical significance was calculated with the paired *t* test (paired data) or unpaired *t*-test (unpaired data). Statistical analysis was performed by Tukey-corrected one-way analysis of variance (ANOVA; multi-group data), or Bonferroni-corrected repeated measures ANOVA (multi-group data at varied time points). A *p* value < 0.05 was regarded as statistically significant.

## Supplementary information

Supplementary Materials

## Data Availability

All the data generated in this study were provided in this article, both as main and supplementary results, and the primary data files are available from the corresponding author upon reasonable request.
