# Supplementary material for: Anti-cancer effect of LINC00478 in bladder cancer correlates with KDM1A-dependent MMP9 demethylation
Source: Cell Death Discov. 2022 May 3;8:242. doi: 10.1038/s41420-022-00956-z (PMC9065159; doi:10.1038/s41420-022-00956-z)
Supplement: Supplementary file 1 — Supplementary Materials [file 41420_2022_956_MOESM1_ESM.docx]

**Supplementary Figure 1.** Representative images of EdU assay in panel 2B (A), wound healing assay in panel 2C (B), Transwell assay in panel 2D (C), and Western blots in panel 2E (D).


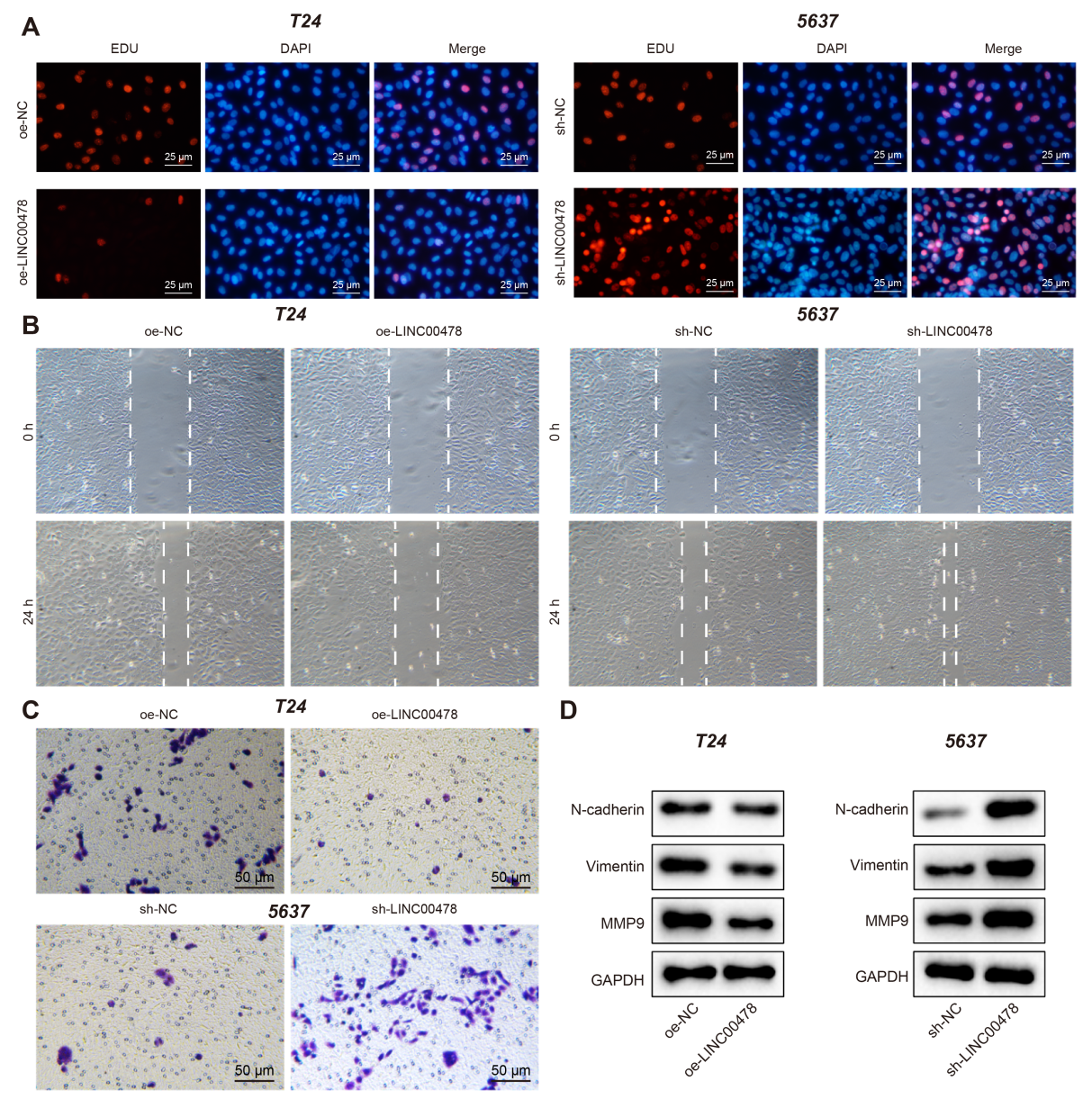


**Supplementary Figure 2** Representative images of Western blots in panel 5B (A), EdU assay in panel 5C (B), wound healing assay in panel 5D (C), and Transwell assay in 5E (D).


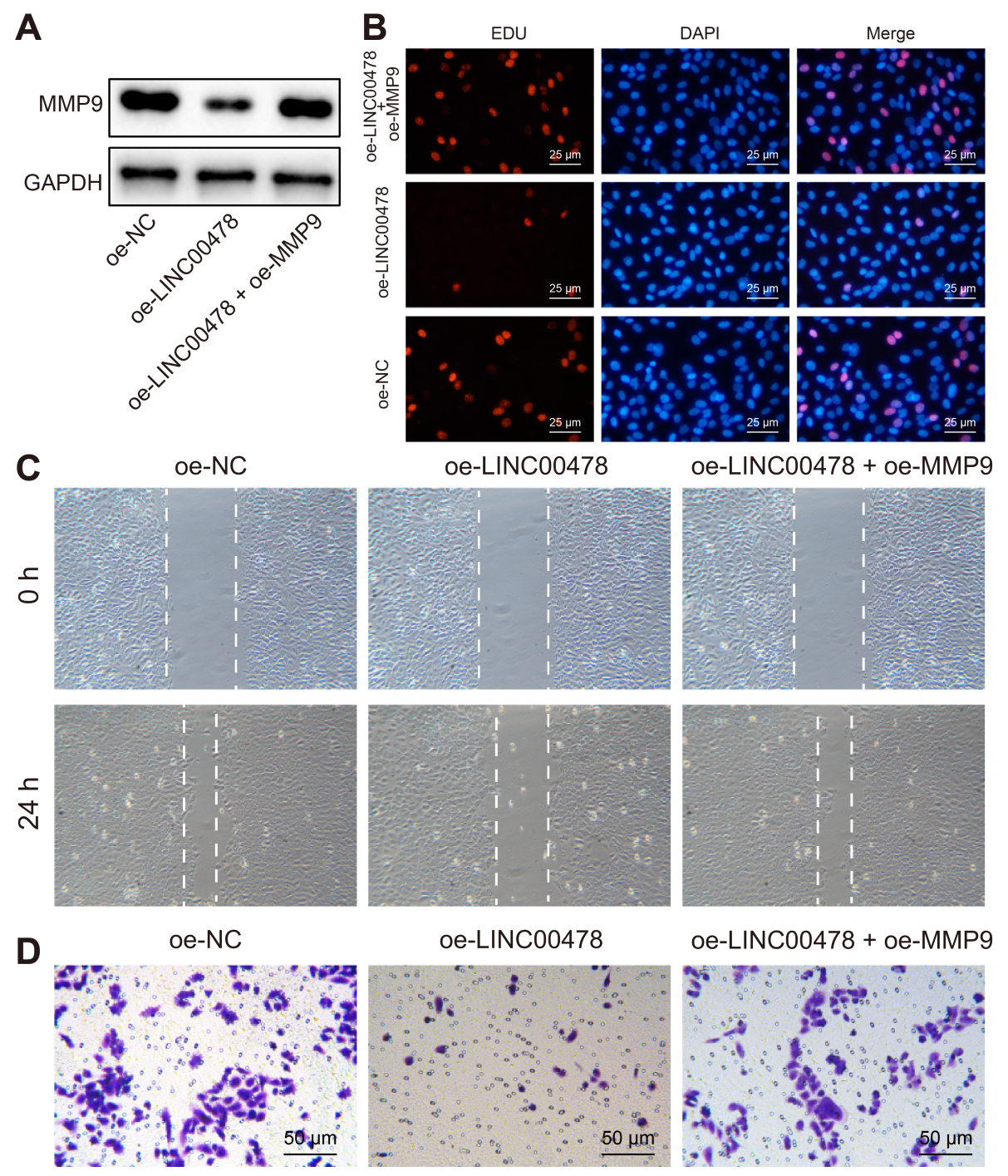


**Supplementary Table 1.** The clinic-pathological factors of 72 patients with bladder cancer

| Characteristics | n | LINC00478 expression | | *p* value |
| --- | --- | --- | --- | --- |
|  |  | Low | High |  |
| Gender |  |  |  | 0.795 |
| Male | 51 | 26 | 25 |  |
| Female | 21 | 10 | 11 |  |
| Age (year) |  |  |  | 0.237 |
| > 60 | 39 | 22 | 17 |  |
| ≤ 60 | 33 | 14 | 19 |  |
| Histological grade |  |  |  | < 0.001 |
| Low | 24 | 20 | 4 |  |
| High | 48 | 16 | 32 |  |
| TNM stage |  |  |  | 0.011 |
| Ι + ΙΙ | 60 | 26 | 34 |  |
| ΙΙΙ | 12 | 10 | 2 |  |
| Lymph node metastasis |  |  |  | 0.317 |
| Yes | 48 | 22 | 26 |  |
| No | 24 | 14 | 10 |  |

Note: The enumeration data was analyzed by the chi-square test. TNM, Tumor, Node, Metastases.

**Supplementary Table 2.** Primer sequences for RT-qPCR

|  |  | Sequence |
| --- | --- | --- |
| LINC00478 | Forward | GCTGGTATTTCACAGCAGCG |
|  | Reverse | CGGATTGGCTGCTAGCTCTT |
| GAPDH | Forward | GAAAGCCTGCCGGTGACTAA |
|  | Reverse | AGGAAAAGCATCACCCGGAG |

Note: RT-qPCR, reverse transcription quantitative polymerase chain reaction; LINC00478, long intergenic non-protein coding RNA 478; GAPDH, glyceraldehyde-3-phosphate dehydrogenase.
